# Monolanthanum tripotassium tetra­hydrogen deca­molybdodicobaltate(III) trideca­hydrate

**DOI:** 10.1107/S1600536810000929

**Published:** 2010-01-23

**Authors:** Uk Lee, Hea-Chung Joo

**Affiliations:** a Department of Chemistry, Pukyong National University, 599-1 Daeyeon 3-dong, Nam-gu, Busan 608-737, Republic of Korea; b Department of Chemistry, Dongeui University, San 24 Kaya-dong Busanjin-gu, Busan 614-714, Republic of Korea

## Abstract

The title compound, K_3_La[H_4_Mo_10_Co_2_O_38_].13H_2_O, is an optically active chiral polyoxometalate (POM) which contains an anion with ideal point symmetry *D*2 (222). The crystals containing one of the enanti­omer pairs in the POM were resolved at pH 2.5. The factor that governs the formation of the compound is the pH condition of the mother liquor. The racemate salt, K_6_[H_4_Mo_10_Co_2_O_38_]·7H_2_O, is obtained at pH 6.5 [Nolan *et al.* (1998 ▶). *Aust. J. Chem.* **51**. 825–834]. Two non-acidic H atoms in the POM form intra­molecular hydrogen bonds and the remaining two H atoms form hydrogen bonds with two water mol­ecules. The POMs are connected by three K^+^ ions. The La^3+^ ion is coordinated by three O atoms of the POM and six water molecues with distances in the range 2.516 (5)–2.589 (5) Å.

## Related literature

For the crystal structures of [H_4_Mo_10_Co_2_O_38_]^6−^, see: Evans & Showell (1969 ▶); Nolan *et al.* (1998 ▶). For the optical resolution, see: Ama *et al.* (1970 ▶). For a review of chirality in POM chemistry, see: Hasenknopf *et al.* (2008 ▶). For bond-valence sum calculations, see: Brown & Altermatt (1985 ▶); Brese & O’Keeffe (1991 ▶).
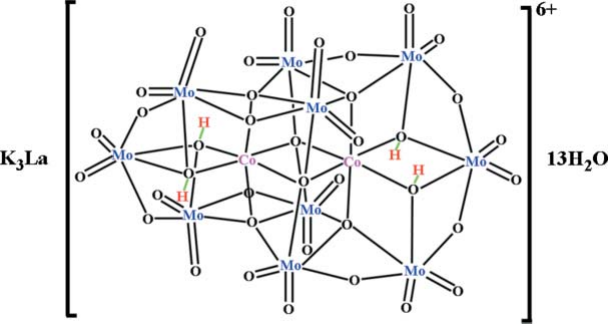


## Experimental

### 

#### Crystal data

K_3_La[H_4_Mo_10_Co_2_O_38_]·13H_2_O
*M* *_r_* = 2179.71
Monoclinic, 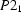

*a* = 10.4487 (6) Å
*b* = 18.598 (1) Å
*c* = 12.3179 (8) Å
β = 112.957 (4)°
*V* = 2204.1 (2) Å^3^
*Z* = 2
Mo *K*α radiation
μ = 4.83 mm^−1^
*T* = 298 K
0.28 × 0.24 × 0.20 mm

#### Data collection

Stoe STADI-4 diffractometer
Absorption correction: numerical (*X-SHAPE*; Stoe & Cie, 1996 ▶) *T*_min_ = 0.349, *T*_max_ = 0.527
10417 measured reflections
10104 independent reflections
9949 reflections with *I* > 2σ(*I*)
*R*_int_ = 0.016
3 standard reflections every 60 min  intensity decay: 2.3%

#### Refinement

*R*[*F* ^2^ > 2σ(*F* ^2^)] = 0.025
*wR*(*F* ^2^) = 0.069
*S* = 1.10
10104 reflections
630 parameters
22 restraints
H atoms treated by a mixture of independent and constrained refinement
Δρ_max_ = 1.37 e Å^−3^
Δρ_min_ = −1.27 e Å^−3^
Absolute structure: Flack (1983 ▶)
Flack parameter: 0.001 (10)

### 

Data collection: *STADI-4* (Stoe & Cie, 1996 ▶); cell refinement: *X-RED* (Stoe & Cie, 1996 ▶); data reduction: *X-RED*; program(s) used to solve structure: *SHELXS97* (Sheldrick, 2008 ▶); program(s) used to refine structure: *SHELXL97* (Sheldrick, 2008 ▶); molecular graphics: *ORTEP-3* (Farrugia, 1997 ▶) and *DIAMOND* (Brandenburg, 1998 ▶); software used to prepare material for publication: *SHELXL97*.

## Supplementary Material

Crystal structure: contains datablocks global, I. DOI: 10.1107/S1600536810000929/br2132sup1.cif

Structure factors: contains datablocks I. DOI: 10.1107/S1600536810000929/br2132Isup2.hkl

Additional supplementary materials:  crystallographic information; 3D view; checkCIF report

## Figures and Tables

**Table 1:** Hydrogen-bond geometry (Å, °)

*D*—H⋯*A*	*D*—H	H⋯*A*	*D*⋯*A*	*D*—H⋯*A*
O7C—H7⋯O30*T*	0.79 (4)	2.16 (5)	2.891 (6)	152 (8)
O8C—H8⋯O9*W*	0.85 (4)	2.26 (6)	2.972 (10)	141 (7)
O9C—H9⋯O8*W*^i^	0.82 (4)	2.21 (6)	2.935 (9)	147 (8)
O10C—H10⋯O21*T*	0.80 (4)	2.27 (6)	2.932 (6)	141 (8)

Symmetry code: (i) 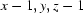.

**Fig. 1.**
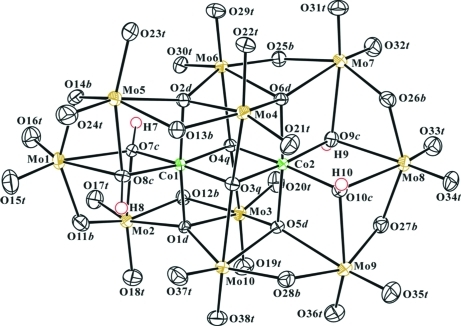
The structure of the [H4Mo10Co2O38]6- POM with the atom numbering scheme. Displacement ellipsoids are drawn at the 50% probability level. H atoms are presented as a small spheres of arbitrary radius.

**Fig. 2.**
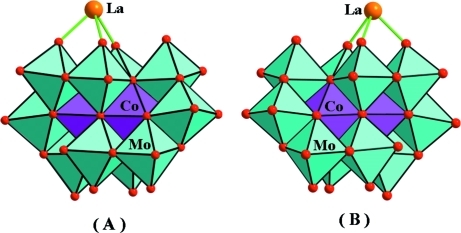
The two enantiomers of the La[Mo10Co2O38]7- POM. The structure described here is (A).

## supplementary crystallographic information

### Comment

The ammonium salt of [H_4_Mo_10_Co_2_O_38_]^6-^ heteropolyoxometalte has been briefly reported as a typical chiral polyoxometalate (POM) (Evans *et al.* 1969) and the crystal strucure of potassium salt, K_6_[H_4_Mo_10_Co_2_O_38_].7H_2_O was reported in detail by P2_1_/c space group (Nolan *et al.* 1998). The study of optical resolution of this POM was carrried out by using [Co(en)_3_]^3+^ (Ama *et al.* 1970). One of the enantiomer pair salts was obtained as crystals. But the crystal structral study of this salt was not carried out. Recently, the micro review of chirality in POM chemistry has been reported (Hasenknopf *et al.* 2008). Somtimes, the lanthanide cation, having a very large oxide affinity and stability at low pH range, is useful in isolating POMs because the lanthanide forms a very stable salt with POMs.

The title compound was obtained as a lantahanide-alkali metal double salt in the monoclinic system in chiral space group P2_1_. Here we report the enantimorphous structure of [H_4_Mo_10_Co_2_O_38_]^6-^ heteropolyoxometalate. The structure of the [H_4_Mo_10_Co_2_O_38_]^6-^ POM (Fig. 1) has been discussed in detail (Nolan *et al.*,  1998). The O atoms are classified in the Fig. 1, *viz*., O*t* (O16-O24, O29-O38;  terminal Mo═O atom), O*b* (O11-O14, O25-O28; O bridged µ_2_-O atom), O*c* (O7-O10; µ_3_-O atom of a Co and two Mo atoms), O*d* (O1, O2, O5, O6; µ_4_-O atom of a Co and three Mo atoms), and O*q* (O3, O4; µ_4_-O atom of two Co and two Mo atoms). Fig. 2 shows the two enantiomers.

The four protonated  O atoms, O*c*(H) in the POM, were identified (Nolan *et al.*,  1998) by calculation of bond-valence sums (BVS; Brown & Altermatt, 1985; Brese & O'Keeffe, 1991). The positions of four non-acidic H atoms on O*c* atoms were found on difference Fourier map in this report. These H atoms formed hydrogen bond intramoleculely and with water molecules (Table. 1). All the water molecules formed hydrogen bonds with O atoms in the polyanions, and there are also O*w*–H···O*w* hydrogen-bond interactions except zeolitic O13*w* molecule. K^+^ ions are coordinated by eight O atoms, *viz*. [K1(O*t*)_7_(O*w*)]^+^, [K2(O*t*)_3_(O*w*)_4_]^+^, and [K3(O*t*)_3_(O*b*)_2_(O*w*)_3_]^+^ in the range 2.69 (1)-3.276 (6) Å.  La^3+^ ion is coordinated by nine O atoms, *viz*. [La(O*t*)_3_(O*w*)_6_]^3+^ in the range 2.516 (5)-2.589 (5) Å.

### Experimental

Crystals of the title compound were obtained from the aqueous solution of La(NO_3_)_3_.6H_2_O and K_6_[H_4_Co_2_Mo_10_O_38_].7H_2_O (Nolan *et al.*,  1998) at pH 2.5.

### Refinement

Four H atoms of [H_4_Co_2_Mo_10_O_38_]^6-^ were positioned in a difference Fourier map and their positional parameters refined with a distance restraint [O–H = 0.85 (5) Å] and these H atoms were refined with an isotropic displacement parameter *U*_iso_ = 1.2*U*_eq_(O). The all H atoms of the water molecules were refined with an isotropic displacement parameter *U*_iso_ = 1.5*U*_eq_(O). The water H atoms in the O1*w* and O12*w* were positioned in a difference Fourier map and their positional parameters refined with a distances restraint [O–H = 0.85 (5) Å]. The water H atoms in the O5*w* were placed in calculated positions. They were included in the refinement of the riding-motion approximation. The water H atoms in the O2*w*, O3*w*, O4*w*, O8*w* and O11*w* were geometrically positioned and refined using a riding model, with O–H = 0.96 Å. The H atoms of the other water molecules were placed in a difference Fourier map and their positional parameters refined with a distances restraint [O–H = 0.85 (5) Å]. The reasonable positions of H atoms in O13*w* molecule could not be obtained by difference Fourier map and riding model because of zeolitic water molecule. The reported Flack parameter [0.001 (10)] was obtained by a TWIN/BASF procedure.

### Crystal data

**Table:**

LaK_3_[H_4_Mo_10_Co_2_O_38_]·13H_2_O	*F*(000) = 2052
*M**_r_* = 2179.71	*D*_x_ = 3.284 Mg m^−^^3^
Monoclinic, *P*2_1_	Mo *K*α radiation, λ = 0.71069 Å
Hall symbol: P 2yb	Cell parameters from 28 reflections
*a* = 10.4487 (6) Å	θ = 19.0–20.9°
*b* = 18.598 (1) Å	µ = 4.83 mm^−^^1^
*c* = 12.3179 (8) Å	*T* = 298 K
β = 112.957 (4)°	Polyhedron, blue
*V* = 2204.1 (2) Å^3^	0.28 × 0.24 × 0.20 mm
*Z* = 2	

### Data collection

**Table:**

Stoe STADI-4 diffractometer	9949 reflections with *I* > 2σ(*I*)
Radiation source: fine-focus sealed tube	*R*_int_ = 0.016
graphite	θ_max_ = 27.5°, θ_min_ = 1.8°
ω/2θ scans	*h* = −13→13
Absorption correction: numerical (*X-SHAPE*; Stoe & Cie, 1996)	*k* = −24→24
*T*_min_ = 0.349, *T*_max_ = 0.527	*l* = −15→15
10417 measured reflections	3 standard reflections every 60 min
10104 independent reflections	intensity decay: 2.3%

### Refinement

**Table:**

Refinement on *F*^2^	Hydrogen site location: difference Fourier map
Least-squares matrix: full	H atoms treated by a mixture of independent and constrained refinement
*R*[*F*^2^ > 2σ(*F*^2^)] = 0.025	*w* = 1/[σ^2^(*F*_o_^2^) + (0.0432*P*)^2^ + 5.9397*P*] where *P* = (*F*_o_^2^ + 2*F*_c_^2^)/3
*wR*(*F*^2^) = 0.069	(Δ/σ)_max_ < 0.001
*S* = 1.10	Δρ_max_ = 1.37 e Å^−^^3^
10104 reflections	Δρ_min_ = −1.27 e Å^−^^3^
630 parameters	Extinction correction: *SHELXL97* (Sheldrick, 2008), Fc^*^=kFc[1+0.001xFc^2^λ^3^/sin(2θ)]^-1/4^
22 restraints	Extinction coefficient: 0.00255 (10)
Primary atom site location: structure-invariant direct methods	Absolute structure: Flack (1983)
Secondary atom site location: difference Fourier map	Flack parameter: 0.001 (10)

### Special details

**Table:**

Geometry. All esds (except the esd in the dihedral angle between two l.s. planes) are estimated using the full covariance matrix. The cell esds are taken into account individually in the estimation of esds in distances, angles and torsion angles; correlations between esds in cell parameters are only used when they are defined by crystal symmetry. An approximate (isotropic) treatment of cell esds is used for estimating esds involving l.s. planes.
Refinement. Refinement of F^2^ against ALL reflections. The weighted R-factor wR and goodness of fit S are based on F^2^, conventional R-factors R are based on F, with F set to zero for negative F^2^. The threshold expression of F^2^ > 2sigma(F^2^) is used only for calculating R-factors(gt) etc. and is not relevant to the choice of reflections for refinement. R-factors based on F^2^ are statistically about twice as large as those based on F, and R- factors based on ALL data will be even larger.

### Fractional atomic coordinates and isotropic or equivalent isotropic displacement parameters (Å^2^)

**Table:**

	*x*	*y*	*z*	*U*_iso_*/*U*_eq_	
La	0.44544 (3)	0.446924 (17)	0.14123 (3)	0.01703 (7)	
Mo1	0.35787 (6)	0.30183 (3)	0.62418 (4)	0.02049 (11)	
Mo2	0.09792 (5)	0.18531 (3)	0.54287 (4)	0.01769 (10)	
Mo3	−0.02214 (5)	0.11683 (3)	0.27366 (4)	0.01671 (10)	
Mo4	0.42999 (5)	0.23358 (2)	0.20358 (4)	0.01407 (9)	
Mo5	0.51945 (5)	0.32853 (3)	0.44537 (4)	0.01653 (10)	
Mo6	0.13311 (5)	0.32304 (2)	0.16909 (4)	0.01389 (9)	
Mo7	0.05653 (5)	0.25070 (3)	−0.09176 (4)	0.01733 (10)	
Mo8	0.06410 (5)	0.07332 (3)	−0.11493 (4)	0.01639 (10)	
Mo9	0.18216 (5)	−0.02480 (3)	0.12967 (4)	0.01894 (10)	
Mo10	0.30682 (5)	0.05651 (2)	0.38835 (4)	0.01552 (9)	
Co1	0.25776 (8)	0.21760 (4)	0.36757 (6)	0.01310 (14)	
Co2	0.16831 (8)	0.15142 (4)	0.14734 (6)	0.01251 (14)	
K1	0.0300 (2)	−0.11545 (10)	0.3699 (2)	0.0461 (4)	
K2	0.6309 (3)	0.28591 (13)	0.9870 (2)	0.0595 (6)	
K3	0.6994 (3)	0.11419 (19)	0.4003 (3)	0.0811 (8)	
O1D	0.1934 (4)	0.1345 (2)	0.4209 (3)	0.0144 (7)	
O2D	0.3143 (4)	0.2948 (2)	0.2990 (3)	0.0152 (8)	
O3Q	0.3316 (4)	0.1562 (2)	0.2853 (4)	0.0165 (8)	
O4Q	0.0974 (4)	0.2140 (2)	0.2295 (3)	0.0148 (7)	
O5D	0.1119 (4)	0.0722 (2)	0.2175 (3)	0.0153 (7)	
O6D	0.2314 (4)	0.2341 (2)	0.0927 (3)	0.0146 (7)	
O7C	0.1753 (4)	0.2738 (2)	0.4538 (3)	0.0154 (7)	
H7	0.130 (7)	0.305 (4)	0.412 (6)	0.023*	
O8C	0.4303 (4)	0.2347 (2)	0.5049 (3)	0.0168 (8)	
H8	0.487 (7)	0.201 (3)	0.512 (7)	0.025*	
O9C	0.0078 (4)	0.1540 (2)	−0.0016 (4)	0.0177 (8)	
H9	−0.073 (5)	0.159 (5)	−0.007 (7)	0.027*	
O10C	0.2394 (4)	0.0811 (2)	0.0682 (4)	0.0164 (8)	
H10	0.312 (6)	0.091 (4)	0.066 (7)	0.025*	
O11B	0.2833 (5)	0.2093 (3)	0.6447 (4)	0.0247 (9)	
O12B	−0.0505 (4)	0.1808 (2)	0.3825 (4)	0.0202 (8)	
O13B	0.5637 (4)	0.2485 (2)	0.3574 (4)	0.0177 (8)	
O14B	0.4073 (5)	0.3666 (2)	0.5211 (4)	0.0217 (8)	
O15T	0.5119 (5)	0.2980 (3)	0.7427 (4)	0.0347 (11)	
O16T	0.2563 (6)	0.3602 (3)	0.6628 (5)	0.0337 (11)	
O17T	0.0054 (6)	0.2421 (3)	0.5929 (4)	0.0316 (11)	
O18T	0.0785 (6)	0.1022 (3)	0.5926 (5)	0.0320 (11)	
O19T	−0.0674 (6)	0.0388 (3)	0.3207 (5)	0.0333 (11)	
O20T	−0.1582 (5)	0.1366 (3)	0.1435 (4)	0.0292 (10)	
O21T	0.4985 (5)	0.1640 (3)	0.1549 (4)	0.0256 (9)	
O22T	0.4651 (4)	0.3102 (2)	0.1398 (4)	0.0202 (8)	
O23T	0.5226 (5)	0.3973 (2)	0.3514 (4)	0.0246 (9)	
O24T	0.6782 (5)	0.3278 (3)	0.5558 (4)	0.0291 (10)	
O25B	−0.0026 (4)	0.3003 (2)	0.0225 (4)	0.0191 (8)	
O26B	0.1374 (4)	0.1659 (2)	−0.1320 (4)	0.0205 (8)	
O27B	0.0277 (4)	0.0062 (2)	−0.0117 (4)	0.0203 (8)	
O28B	0.3425 (5)	0.0027 (2)	0.2733 (4)	0.0215 (9)	
O29T	0.2215 (4)	0.3920 (2)	0.1336 (4)	0.0223 (9)	
O30T	0.0460 (5)	0.3621 (2)	0.2463 (4)	0.0228 (9)	
O31T	0.1538 (5)	0.3148 (2)	−0.1229 (4)	0.0260 (9)	
O32T	−0.1007 (5)	0.2530 (3)	−0.2044 (4)	0.0298 (10)	
O33T	−0.0996 (5)	0.0761 (3)	−0.2239 (4)	0.0278 (10)	
O34T	0.1615 (5)	0.0218 (3)	−0.1690 (4)	0.0258 (9)	
O35T	0.2728 (6)	−0.0787 (3)	0.0729 (5)	0.0336 (11)	
O36T	0.0954 (6)	−0.0806 (3)	0.1858 (5)	0.0334 (11)	
O37T	0.4685 (5)	0.0749 (3)	0.4933 (4)	0.0279 (10)	
O38T	0.2395 (5)	−0.0078 (3)	0.4493 (4)	0.0264 (9)	
O1W	0.3980 (6)	0.3956 (3)	−0.0655 (4)	0.0354 (12)	
H1A	0.429 (11)	0.419 (5)	−0.117 (8)	0.053*	
H1B	0.327 (9)	0.373 (5)	−0.060 (9)	0.053*	
O2W	0.5027 (5)	0.5444 (3)	0.0224 (4)	0.0336 (11)	
H2A	0.5342	0.5939	0.0474	0.050*	
H2B	0.4543	0.5348	−0.0603	0.050*	
O3W	0.6426 (6)	0.5253 (3)	0.2868 (5)	0.0436 (14)	
H3A	0.7189	0.4949	0.3327	0.065*	
H3B	0.6734	0.5593	0.2435	0.065*	
O4W	0.2314 (5)	0.5129 (3)	0.0086 (5)	0.0403 (13)	
H4A	0.2581	0.5535	−0.0260	0.060*	
H4B	0.1804	0.5293	0.0540	0.060*	
O5W	0.6810 (5)	0.3994 (3)	0.1512 (5)	0.0337 (11)	
H5A	0.7359	0.3822	0.2301	0.051*	
H5B	0.7321	0.4376	0.1325	0.051*	
O6W	0.3676 (6)	0.5322 (4)	0.2677 (5)	0.0470 (16)	
H6A	0.4185	0.5505	0.3236	0.071*	
H6B	0.2906	0.5318	0.2551	0.071*	
O7W	0.0072 (8)	−0.0501 (4)	0.5720 (7)	0.067 (2)	
H7A	0.0214	−0.0456	0.6523	0.101*	
H7B	0.0600	−0.0188	0.5680	0.101*	
O8W	0.7068 (8)	0.1744 (5)	0.8755 (8)	0.069 (2)	
H8A	0.7537	0.1937	0.8288	0.104*	
H8B	0.6251	0.1489	0.8255	0.104*	
O9W	0.7144 (9)	0.1721 (4)	0.6059 (9)	0.115 (5)	
H9A	0.7267	0.2218	0.6169	0.172*	
H9B	0.7625	0.1503	0.6639	0.172*	
O10W	0.7381 (9)	−0.0065 (4)	0.5288 (12)	0.110 (5)	
H10A	0.8166	−0.0032	0.5439	0.165*	
H10B	0.7127	0.0026	0.5759	0.165*	
O11W	0.4099 (6)	0.1777 (3)	0.8878 (5)	0.0381 (12)	
H11A	0.3306	0.1915	0.9041	0.057*	
H11C	0.3838	0.1748	0.8041	0.057*	
O12W	0.5854 (7)	−0.0010 (4)	0.2258 (6)	0.0480 (15)	
H12A	0.515 (7)	−0.010 (7)	0.246 (10)	0.072*	
H12B	0.650 (8)	−0.027 (6)	0.290 (8)	0.072*	
O13W	0.8073 (8)	0.3096 (4)	0.3319 (9)	0.077 (3)	

### Atomic displacement parameters (Å^2^)

**Table:**

	*U*^11^	*U*^22^	*U*^33^	*U*^12^	*U*^13^	*U*^23^
La	0.01867 (15)	0.01501 (14)	0.01674 (15)	−0.00193 (12)	0.00619 (11)	0.00159 (11)
Mo1	0.0262 (3)	0.0214 (2)	0.0125 (2)	−0.00452 (19)	0.00618 (19)	−0.00344 (18)
Mo2	0.0230 (2)	0.0174 (2)	0.0143 (2)	−0.00067 (18)	0.00903 (18)	0.00012 (17)
Mo3	0.0166 (2)	0.0177 (2)	0.0167 (2)	−0.00258 (17)	0.00736 (18)	−0.00267 (17)
Mo4	0.0144 (2)	0.0130 (2)	0.0148 (2)	−0.00060 (16)	0.00567 (16)	0.00003 (16)
Mo5	0.0182 (2)	0.0155 (2)	0.0132 (2)	−0.00354 (17)	0.00326 (17)	0.00012 (17)
Mo6	0.0151 (2)	0.0118 (2)	0.0142 (2)	0.00074 (16)	0.00515 (16)	0.00089 (16)
Mo7	0.0207 (2)	0.0162 (2)	0.0136 (2)	0.00059 (17)	0.00507 (17)	0.00143 (17)
Mo8	0.0162 (2)	0.0170 (2)	0.0149 (2)	0.00093 (17)	0.00489 (17)	−0.00296 (17)
Mo9	0.0241 (2)	0.0126 (2)	0.0193 (2)	−0.00084 (18)	0.00764 (19)	−0.00130 (17)
Mo10	0.0178 (2)	0.0132 (2)	0.0144 (2)	0.00079 (17)	0.00502 (16)	0.00198 (16)
Co1	0.0160 (3)	0.0119 (3)	0.0106 (3)	−0.0016 (3)	0.0043 (3)	−0.0003 (2)
Co2	0.0148 (3)	0.0104 (3)	0.0115 (3)	−0.0005 (2)	0.0043 (3)	−0.0003 (2)
K1	0.0452 (10)	0.0339 (9)	0.0664 (12)	−0.0074 (7)	0.0297 (9)	0.0064 (8)
K2	0.0837 (16)	0.0500 (12)	0.0427 (11)	−0.0020 (11)	0.0224 (11)	−0.0147 (9)
K3	0.0781 (18)	0.0793 (19)	0.087 (2)	0.0011 (15)	0.0335 (16)	−0.0015 (16)
O1D	0.022 (2)	0.0108 (17)	0.0138 (17)	−0.0020 (14)	0.0103 (15)	−0.0014 (14)
O2D	0.0138 (18)	0.0146 (18)	0.0137 (18)	−0.0019 (14)	0.0015 (15)	0.0010 (14)
O3Q	0.021 (2)	0.0143 (18)	0.0153 (18)	0.0000 (15)	0.0076 (16)	0.0013 (14)
O4Q	0.0154 (18)	0.0138 (18)	0.0129 (17)	−0.0014 (14)	0.0033 (14)	0.0004 (14)
O5D	0.0175 (18)	0.0121 (18)	0.0160 (18)	−0.0011 (14)	0.0063 (15)	0.0002 (14)
O6D	0.0160 (17)	0.0128 (17)	0.0127 (17)	−0.0025 (15)	0.0031 (14)	0.0015 (14)
O7C	0.021 (2)	0.0117 (17)	0.0128 (18)	−0.0010 (15)	0.0053 (15)	−0.0004 (14)
O8C	0.0208 (19)	0.0126 (18)	0.0155 (18)	0.0023 (15)	0.0053 (15)	0.0035 (14)
O9C	0.0156 (18)	0.019 (2)	0.0180 (19)	−0.0010 (16)	0.0059 (16)	−0.0024 (15)
O10C	0.0157 (18)	0.0152 (18)	0.0175 (19)	−0.0008 (15)	0.0056 (15)	−0.0008 (15)
O11B	0.027 (2)	0.027 (2)	0.019 (2)	−0.0025 (18)	0.0072 (18)	0.0035 (17)
O12B	0.021 (2)	0.021 (2)	0.019 (2)	0.0033 (16)	0.0083 (16)	−0.0012 (16)
O13B	0.0154 (18)	0.018 (2)	0.0174 (19)	−0.0010 (15)	0.0043 (15)	−0.0003 (15)
O14B	0.033 (2)	0.0134 (18)	0.022 (2)	−0.0033 (17)	0.0131 (18)	−0.0028 (16)
O15T	0.036 (3)	0.043 (3)	0.020 (2)	−0.010 (2)	0.006 (2)	−0.006 (2)
O16T	0.042 (3)	0.033 (3)	0.031 (2)	−0.001 (2)	0.020 (2)	−0.006 (2)
O17T	0.043 (3)	0.029 (3)	0.029 (2)	0.005 (2)	0.020 (2)	−0.005 (2)
O18T	0.041 (3)	0.027 (2)	0.031 (2)	−0.005 (2)	0.017 (2)	0.004 (2)
O19T	0.040 (3)	0.025 (2)	0.041 (3)	−0.004 (2)	0.022 (2)	0.001 (2)
O20T	0.019 (2)	0.039 (3)	0.026 (2)	0.0009 (18)	0.0043 (18)	−0.005 (2)
O21T	0.029 (2)	0.021 (2)	0.029 (2)	−0.0005 (18)	0.0133 (19)	−0.0043 (17)
O22T	0.022 (2)	0.020 (2)	0.021 (2)	−0.0010 (16)	0.0103 (17)	0.0050 (16)
O23T	0.028 (2)	0.021 (2)	0.021 (2)	−0.0080 (18)	0.0054 (18)	−0.0030 (17)
O24T	0.027 (2)	0.026 (2)	0.027 (2)	−0.0026 (19)	0.0034 (19)	0.0036 (19)
O25B	0.0191 (19)	0.0190 (19)	0.0174 (19)	0.0049 (16)	0.0052 (16)	0.0001 (16)
O26B	0.023 (2)	0.022 (2)	0.019 (2)	0.0008 (17)	0.0111 (17)	0.0024 (16)
O27B	0.020 (2)	0.020 (2)	0.020 (2)	−0.0043 (16)	0.0071 (16)	−0.0037 (16)
O28B	0.022 (2)	0.018 (2)	0.021 (2)	0.0026 (16)	0.0056 (17)	0.0000 (16)
O29T	0.020 (2)	0.020 (2)	0.024 (2)	0.0011 (16)	0.0055 (17)	0.0069 (17)
O30T	0.029 (2)	0.018 (2)	0.023 (2)	0.0017 (17)	0.0121 (18)	−0.0042 (17)
O31T	0.031 (2)	0.020 (2)	0.030 (2)	−0.0030 (18)	0.0149 (19)	0.0047 (18)
O32T	0.030 (2)	0.030 (2)	0.021 (2)	0.005 (2)	0.0013 (18)	0.0008 (19)
O33T	0.025 (2)	0.030 (2)	0.025 (2)	0.0004 (19)	0.0060 (18)	−0.0020 (19)
O34T	0.033 (2)	0.023 (2)	0.025 (2)	0.0072 (19)	0.0155 (19)	−0.0044 (18)
O35T	0.043 (3)	0.027 (2)	0.031 (3)	0.009 (2)	0.016 (2)	−0.005 (2)
O36T	0.043 (3)	0.022 (2)	0.033 (3)	−0.007 (2)	0.013 (2)	0.000 (2)
O37T	0.026 (2)	0.030 (2)	0.022 (2)	0.0021 (19)	0.0039 (18)	0.0057 (18)
O38T	0.033 (2)	0.020 (2)	0.028 (2)	0.0039 (18)	0.014 (2)	0.0063 (18)
O1W	0.049 (3)	0.036 (3)	0.020 (2)	−0.013 (2)	0.012 (2)	−0.003 (2)
O2W	0.033 (3)	0.035 (3)	0.029 (2)	−0.011 (2)	0.008 (2)	0.006 (2)
O3W	0.042 (3)	0.053 (4)	0.034 (3)	−0.025 (3)	0.014 (2)	−0.016 (3)
O4W	0.024 (2)	0.046 (3)	0.047 (3)	0.006 (2)	0.010 (2)	0.027 (3)
O5W	0.030 (2)	0.033 (3)	0.042 (3)	−0.008 (2)	0.017 (2)	0.003 (2)
O6W	0.028 (3)	0.061 (4)	0.042 (3)	0.008 (3)	0.003 (2)	−0.025 (3)
O7W	0.080 (5)	0.054 (4)	0.056 (4)	−0.028 (4)	0.013 (4)	0.006 (4)
O8W	0.062 (5)	0.072 (5)	0.089 (6)	0.009 (4)	0.045 (4)	0.015 (5)
O9W	0.086 (6)	0.044 (4)	0.118 (8)	−0.021 (4)	−0.064 (6)	0.034 (5)
O10W	0.055 (5)	0.031 (4)	0.231 (14)	−0.005 (3)	0.040 (7)	−0.025 (6)
O11W	0.040 (3)	0.046 (3)	0.033 (3)	0.009 (2)	0.019 (2)	0.012 (2)
O12W	0.038 (3)	0.052 (4)	0.054 (4)	−0.001 (3)	0.018 (3)	−0.015 (3)
O13W	0.050 (4)	0.061 (5)	0.128 (7)	0.027 (4)	0.045 (5)	0.050 (5)

### Geometric parameters (Å, °)

**Table:**

Mo1—Mo5	3.2938 (7)	Mo6—O30T	1.714 (4)
Mo1—Mo2	3.3098 (7)	Mo6—O29T	1.734 (4)
Mo1—Co1	3.3106 (9)	Mo6—O25B	1.858 (4)
Mo2—Co1	3.2599 (9)	Mo6—O2D	2.014 (4)
Mo2—Mo3	3.3086 (7)	Mo6—O4Q	2.240 (4)
Mo3—Co2	3.0348 (9)	Mo6—O6D	2.330 (4)
Mo3—Co1	3.2809 (9)	Mo7—O32T	1.688 (5)
Mo3—Mo10	3.3586 (7)	Mo7—O31T	1.705 (4)
Mo3—Mo9	4.1946 (7)	Mo7—O26B	1.943 (4)
Mo4—O21T	1.697 (5)	Mo7—O25B	1.973 (4)
Mo4—O22T	1.734 (4)	Mo7—O9C	2.273 (4)
Mo4—O13B	1.885 (4)	Mo7—O6D	2.314 (4)
Mo4—O6D	1.991 (4)	Mo8—O34T	1.710 (4)
Mo4—O3Q	2.224 (4)	Mo8—O33T	1.714 (5)
Mo4—O2D	2.290 (4)	Mo8—O27B	1.922 (4)
Mo4—Co2	2.9687 (9)	Mo8—O26B	1.928 (4)
Mo4—Co1	3.2004 (9)	Mo8—O9C	2.278 (4)
Mo4—Mo6	3.3975 (7)	Mo8—O10C	2.290 (4)
Mo5—Co1	3.2569 (9)	Mo9—O36T	1.693 (5)
Mo5—Mo6	4.1438 (7)	Mo9—O35T	1.705 (5)
Mo5—Co2	5.2232 (9)	Mo9—O27B	1.944 (4)
Mo6—Co1	3.0057 (8)	Mo9—O28B	1.972 (4)
Mo6—Co2	3.2359 (9)	Mo9—O10C	2.271 (4)
Mo6—Mo7	3.2816 (7)	Mo9—O5D	2.363 (4)
Mo7—Co2	3.2810 (9)	Mo10—O38T	1.704 (5)
Mo8—Co2	3.3148 (8)	Mo10—O37T	1.714 (5)
Mo8—Mo9	3.3213 (7)	Mo10—O28B	1.887 (4)
Mo9—Co2	3.2915 (9)	Mo10—O1D	2.009 (4)
Mo9—Mo10	3.3014 (7)	Mo10—O5D	2.306 (4)
Mo10—Co1	3.0334 (9)	Mo10—O3Q	2.318 (4)
Mo10—Co2	3.2667 (9)	Co1—O4Q	1.866 (4)
Co1—Co2	2.7874 (10)	Co1—O2D	1.874 (4)
La—O4W	2.516 (5)	Co1—O3Q	1.880 (4)
La—O29T	2.521 (4)	Co1—O1D	1.902 (4)
La—O2W	2.543 (5)	Co1—O7C	1.918 (4)
La—O22T	2.552 (4)	Co1—O8C	1.958 (4)
La—O23T	2.564 (5)	Co2—O4Q	1.874 (4)
La—O6W	2.567 (5)	Co2—O3Q	1.883 (4)
La—O5W	2.572 (5)	Co2—O6D	1.897 (4)
La—O1W	2.579 (5)	Co2—O5D	1.913 (4)
La—O3W	2.589 (5)	Co2—O10C	1.944 (4)
Mo1—O15T	1.702 (5)	Co2—O9C	1.944 (4)
Mo1—O16T	1.711 (5)	K1—O36T	2.690 (6)
Mo1—O11B	1.946 (5)	K1—O17T^i^	2.739 (5)
Mo1—O14B	1.960 (4)	K1—O38T	2.843 (5)
Mo1—O8C	2.271 (4)	K1—O7W	2.863 (9)
Mo1—O7C	2.279 (4)	K1—O16T^i^	2.894 (6)
Mo2—O17T	1.701 (5)	K1—O24T^ii^	3.011 (5)
Mo2—O18T	1.704 (5)	K1—O19T	3.025 (6)
Mo2—O11B	1.905 (5)	K1—O31T^iii^	3.174 (5)
Mo2—O12B	1.981 (4)	K2—O8W	2.770 (9)
Mo2—O7C	2.293 (4)	K2—O15T	2.781 (5)
Mo2—O1D	2.306 (4)	K2—O5W^iv^	2.827 (6)
Mo3—O19T	1.697 (5)	K2—O35T^v^	2.912 (5)
Mo3—O20T	1.717 (5)	K2—O11W	2.945 (7)
Mo3—O12B	1.900 (4)	K2—O1W^iv^	3.046 (7)
Mo3—O5D	1.971 (4)	K2—O22T^iv^	3.048 (5)
Mo3—O1D	2.299 (4)	K3—O10W	2.685 (12)
Mo3—O4Q	2.378 (4)	K3—O9W	2.700 (13)
Mo5—O24T	1.686 (5)	K3—O13B	2.819 (5)
Mo5—O23T	1.734 (5)	K3—O12W	2.939 (7)
Mo5—O14B	1.898 (4)	K3—O12B^vi^	2.977 (5)
Mo5—O13B	1.998 (4)	K3—O21T	3.075 (6)
Mo5—O8C	2.232 (4)	K3—O37T	3.134 (6)
Mo5—O2D	2.286 (4)	K3—O19T^vi^	3.276 (6)
			
Co2—Mo3—Mo9	51.149 (17)	O34T—Mo8—O26B	97.8 (2)
Co1—Mo3—Mo9	88.689 (18)	O33T—Mo8—O26B	101.8 (2)
Mo2—Mo3—Mo9	127.515 (17)	O27B—Mo8—O26B	147.24 (18)
Mo10—Mo3—Mo9	50.358 (13)	O34T—Mo8—O9C	160.5 (2)
Co2—Mo4—Mo6	60.658 (18)	O33T—Mo8—O9C	92.4 (2)
Co1—Mo4—Mo6	54.102 (17)	O27B—Mo8—O9C	81.79 (17)
Co1—Mo5—Mo6	46.018 (15)	O26B—Mo8—O9C	71.78 (17)
Mo1—Mo5—Mo6	87.491 (16)	O34T—Mo8—O10C	92.88 (19)
Co1—Mo6—Mo7	113.07 (2)	O33T—Mo8—O10C	160.05 (19)
Co2—Mo6—Mo7	60.447 (17)	O27B—Mo8—O10C	71.20 (16)
Co1—Mo6—Mo5	51.231 (17)	O26B—Mo8—O10C	81.51 (16)
Co2—Mo6—Mo5	89.230 (18)	O9C—Mo8—O10C	69.71 (15)
Mo7—Mo6—Mo5	126.989 (17)	O36T—Mo9—O35T	106.2 (3)
Mo4—Mo6—Mo5	50.202 (12)	O36T—Mo9—O27B	99.1 (2)
Co2—Mo8—Mo9	59.471 (17)	O35T—Mo9—O27B	101.9 (2)
Co2—Mo9—Mo8	60.167 (17)	O36T—Mo9—O28B	101.3 (2)
Mo10—Mo9—Mo8	119.405 (19)	O35T—Mo9—O28B	96.2 (2)
Co2—Mo9—Mo3	45.892 (16)	O27B—Mo9—O28B	147.68 (18)
Mo10—Mo9—Mo3	51.573 (13)	O36T—Mo9—O10C	156.9 (2)
Mo8—Mo9—Mo3	88.185 (16)	O35T—Mo9—O10C	96.4 (2)
Co1—Mo10—Mo3	61.518 (18)	O27B—Mo9—O10C	71.25 (16)
Co2—Mo10—Mo3	54.502 (17)	O28B—Mo9—O10C	80.34 (17)
Mo9—Mo10—Mo3	78.068 (16)	O36T—Mo9—O5D	88.2 (2)
Mo6—Co1—Mo10	135.92 (3)	O35T—Mo9—O5D	163.3 (2)
Co2—Co1—Mo5	119.37 (3)	O27B—Mo9—O5D	83.44 (16)
Mo6—Co1—Mo5	82.75 (2)	O28B—Mo9—O5D	72.42 (16)
Mo10—Co1—Mo5	120.40 (3)	O10C—Mo9—O5D	70.10 (14)
Mo4—Co1—Mo5	60.838 (18)	O38T—Mo10—O37T	105.2 (2)
Co2—Co1—Mo2	119.96 (3)	O38T—Mo10—O28B	101.3 (2)
Mo6—Co1—Mo2	119.18 (3)	O37T—Mo10—O28B	104.3 (2)
Mo10—Co1—Mo2	82.85 (2)	O38T—Mo10—O1D	92.40 (19)
Mo4—Co1—Mo2	174.10 (3)	O37T—Mo10—O1D	101.0 (2)
Mo5—Co1—Mo2	120.67 (2)	O28B—Mo10—O1D	146.84 (17)
Co1—Co2—Mo3	68.45 (2)	O38T—Mo10—O5D	97.01 (19)
Mo4—Co2—Mo3	135.88 (3)	O37T—Mo10—O5D	157.32 (19)
Co1—Co2—Mo6	59.31 (2)	O28B—Mo10—O5D	75.21 (16)
Mo4—Co2—Mo6	66.238 (19)	O1D—Mo10—O5D	73.20 (15)
Mo3—Co2—Mo6	92.76 (2)	O38T—Mo10—O3Q	163.06 (19)
Co1—Co2—Mo10	59.51 (2)	O37T—Mo10—O3Q	88.05 (19)
Mo4—Co2—Mo10	91.42 (2)	O28B—Mo10—O3Q	85.20 (17)
Mo3—Co2—Mo10	64.292 (19)	O1D—Mo10—O3Q	74.46 (15)
Mo6—Co2—Mo10	118.82 (2)	O5D—Mo10—O3Q	69.28 (14)
Co1—Co2—Mo7	119.47 (3)	O4Q—Co1—O2D	86.85 (17)
Mo4—Co2—Mo7	83.84 (2)	O4Q—Co1—O3Q	84.16 (17)
Mo3—Co2—Mo7	120.30 (3)	O2D—Co1—O3Q	87.36 (18)
Mo6—Co2—Mo7	60.465 (17)	O4Q—Co1—O1D	88.47 (18)
Mo10—Co2—Mo7	175.05 (3)	O2D—Co1—O1D	173.81 (17)
O4W—La—O29T	66.17 (15)	O3Q—Co1—O1D	88.13 (17)
O4W—La—O2W	68.13 (17)	O4Q—Co1—O7C	94.97 (18)
O29T—La—O2W	133.65 (15)	O2D—Co1—O7C	96.95 (17)
O4W—La—O22T	122.37 (17)	O3Q—Co1—O7C	175.56 (18)
O29T—La—O22T	70.86 (14)	O1D—Co1—O7C	87.49 (17)
O2W—La—O22T	131.96 (16)	O4Q—Co1—O8C	171.94 (17)
O4W—La—O23T	132.93 (17)	O2D—Co1—O8C	85.36 (16)
O29T—La—O23T	79.26 (14)	O3Q—Co1—O8C	97.53 (18)
O2W—La—O23T	141.80 (15)	O1D—Co1—O8C	99.44 (17)
O22T—La—O23T	69.73 (14)	O7C—Co1—O8C	83.94 (17)
O4W—La—O6W	71.4 (2)	O4Q—Co2—O3Q	83.85 (17)
O29T—La—O6W	76.34 (18)	O4Q—Co2—O6D	87.29 (17)
O2W—La—O6W	96.3 (2)	O3Q—Co2—O6D	87.93 (17)
O22T—La—O6W	131.68 (19)	O4Q—Co2—O5D	88.76 (17)
O23T—La—O6W	70.06 (18)	O3Q—Co2—O5D	87.67 (17)
O4W—La—O5W	142.23 (18)	O6D—Co2—O5D	174.38 (17)
O29T—La—O5W	136.00 (15)	O4Q—Co2—O10C	176.06 (18)
O2W—La—O5W	80.62 (17)	O3Q—Co2—O10C	95.49 (18)
O22T—La—O5W	65.16 (15)	O6D—Co2—O10C	96.57 (17)
O23T—La—O5W	84.83 (16)	O5D—Co2—O10C	87.33 (17)
O6W—La—O5W	134.97 (17)	O4Q—Co2—O9C	96.70 (17)
O4W—La—O1W	76.1 (2)	O3Q—Co2—O9C	174.21 (19)
O29T—La—O1W	89.71 (17)	O6D—Co2—O9C	86.34 (17)
O2W—La—O1W	72.13 (18)	O5D—Co2—O9C	98.10 (18)
O22T—La—O1W	67.04 (16)	O10C—Co2—O9C	84.35 (17)
O23T—La—O1W	136.64 (16)	O36T—K1—O38T	72.89 (15)
O6W—La—O1W	147.46 (19)	O36T—K1—O7W	139.94 (19)
O5W—La—O1W	74.47 (19)	O38T—K1—O7W	73.50 (18)
O4W—La—O3W	116.2 (2)	O36T—K1—O19T	77.21 (16)
O29T—La—O3W	136.91 (17)	O38T—K1—O19T	63.57 (14)
O2W—La—O3W	72.26 (18)	O7W—K1—O19T	68.66 (19)
O22T—La—O3W	121.42 (18)	O8W—K2—O15T	67.5 (2)
O23T—La—O3W	69.62 (17)	O8W—K2—O11W	67.2 (2)
O6W—La—O3W	65.58 (19)	O15T—K2—O11W	70.08 (16)
O5W—La—O3W	70.87 (19)	O10W—K3—O9W	80.8 (3)
O1W—La—O3W	133.36 (18)	O10W—K3—O13B	143.2 (3)
O15T—Mo1—O16T	105.6 (3)	O9W—K3—O13B	70.32 (19)
O15T—Mo1—O11B	98.3 (2)	O10W—K3—O12W	75.3 (3)
O16T—Mo1—O11B	101.7 (2)	O9W—K3—O12W	146.7 (3)
O15T—Mo1—O14B	100.4 (2)	O13B—K3—O12W	118.57 (19)
O16T—Mo1—O14B	98.0 (2)	O10W—K3—O21T	136.0 (3)
O11B—Mo1—O14B	148.00 (18)	O9W—K3—O21T	124.7 (2)
O15T—Mo1—O8C	95.2 (2)	O13B—K3—O21T	56.49 (13)
O16T—Mo1—O8C	158.2 (2)	O12W—K3—O21T	64.78 (18)
O11B—Mo1—O8C	81.34 (18)	O10W—K3—O37T	63.2 (2)
O14B—Mo1—O8C	71.38 (16)	O9W—K3—O37T	61.4 (2)
O15T—Mo1—O7C	162.4 (2)	O13B—K3—O37T	82.69 (15)
O16T—Mo1—O7C	90.8 (2)	O12W—K3—O37T	86.96 (18)
O11B—Mo1—O7C	71.57 (17)	Co1—O1D—Mo10	101.68 (17)
O14B—Mo1—O7C	83.18 (16)	Co1—O1D—Mo3	102.32 (17)
O8C—Mo1—O7C	69.45 (14)	Mo10—O1D—Mo3	102.24 (16)
O17T—Mo2—O18T	105.6 (3)	Co1—O1D—Mo2	101.13 (16)
O17T—Mo2—O11B	101.0 (2)	Mo10—O1D—Mo2	149.8 (2)
O18T—Mo2—O11B	102.1 (2)	Mo3—O1D—Mo2	91.87 (14)
O17T—Mo2—O12B	93.1 (2)	Co1—O2D—Mo6	101.18 (17)
O18T—Mo2—O12B	99.4 (2)	Co1—O2D—Mo5	102.60 (16)
O11B—Mo2—O12B	149.98 (18)	Mo6—O2D—Mo5	148.9 (2)
O17T—Mo2—O7C	94.8 (2)	Co1—O2D—Mo4	99.97 (17)
O18T—Mo2—O7C	159.6 (2)	Mo6—O2D—Mo4	104.06 (16)
O11B—Mo2—O7C	71.90 (17)	Mo5—O2D—Mo4	91.22 (14)
O12B—Mo2—O7C	80.67 (16)	Co1—O3Q—Co2	95.60 (18)
O17T—Mo2—O1D	160.4 (2)	Co1—O3Q—Mo4	102.17 (18)
O18T—Mo2—O1D	90.3 (2)	Co2—O3Q—Mo4	92.20 (16)
O11B—Mo2—O1D	86.44 (18)	Co1—O3Q—Mo10	91.93 (16)
O12B—Mo2—O1D	72.67 (16)	Co2—O3Q—Mo10	101.58 (18)
O7C—Mo2—O1D	70.10 (14)	Mo4—O3Q—Mo10	159.3 (2)
O19T—Mo3—O20T	105.2 (3)	Co1—O4Q—Co2	96.37 (18)
O19T—Mo3—O12B	98.9 (2)	Co1—O4Q—Mo6	93.67 (16)
O20T—Mo3—O12B	103.0 (2)	Co2—O4Q—Mo6	103.37 (18)
O19T—Mo3—O5D	95.2 (2)	Co1—O4Q—Mo3	100.57 (17)
O20T—Mo3—O5D	101.8 (2)	Co2—O4Q—Mo3	90.27 (15)
O12B—Mo3—O5D	147.02 (18)	Mo6—O4Q—Mo3	159.10 (19)
O19T—Mo3—O1D	99.8 (2)	Co2—O5D—Mo3	102.77 (18)
O20T—Mo3—O1D	154.9 (2)	Co2—O5D—Mo10	101.07 (17)
O12B—Mo3—O1D	74.24 (16)	Mo3—O5D—Mo10	103.20 (17)
O5D—Mo3—O1D	74.05 (15)	Co2—O5D—Mo9	100.13 (17)
O19T—Mo3—O4Q	165.8 (2)	Mo3—O5D—Mo9	150.7 (2)
O20T—Mo3—O4Q	86.6 (2)	Mo10—O5D—Mo9	89.97 (14)
O12B—Mo3—O4Q	85.74 (16)	Co2—O6D—Mo4	99.54 (17)
O5D—Mo3—O4Q	74.38 (15)	Co2—O6D—Mo7	101.89 (16)
O1D—Mo3—O4Q	68.36 (13)	Mo4—O6D—Mo7	152.5 (2)
O21T—Mo4—O22T	105.9 (2)	Co2—O6D—Mo6	99.41 (17)
O21T—Mo4—O13B	102.6 (2)	Mo4—O6D—Mo6	103.42 (16)
O22T—Mo4—O13B	97.43 (19)	Mo7—O6D—Mo6	89.92 (14)
O21T—Mo4—O6D	103.4 (2)	Co1—O7C—Mo1	103.82 (18)
O22T—Mo4—O6D	91.90 (18)	Co1—O7C—Mo2	101.06 (17)
O13B—Mo4—O6D	148.71 (17)	Mo1—O7C—Mo2	92.75 (14)
O21T—Mo4—O3Q	90.01 (19)	Co1—O7C—H7	109 (6)
O22T—Mo4—O3Q	162.41 (17)	Mo1—O7C—H7	120 (6)
O13B—Mo4—O3Q	86.14 (16)	Mo2—O7C—H7	126 (6)
O6D—Mo4—O3Q	76.79 (15)	Co1—O8C—Mo5	101.83 (17)
O21T—Mo4—O2D	160.03 (19)	Co1—O8C—Mo1	102.79 (18)
O22T—Mo4—O2D	94.07 (17)	Mo5—O8C—Mo1	94.00 (15)
O13B—Mo4—O2D	75.14 (16)	Co1—O8C—H8	110 (6)
O6D—Mo4—O2D	74.45 (15)	Mo5—O8C—H8	105 (6)
O3Q—Mo4—O2D	70.10 (15)	Mo1—O8C—H8	137 (6)
O24T—Mo5—O23T	106.0 (2)	Co2—O9C—Mo7	101.86 (18)
O24T—Mo5—O14B	102.0 (2)	Co2—O9C—Mo8	103.17 (18)
O23T—Mo5—O14B	103.2 (2)	Mo7—O9C—Mo8	93.49 (16)
O24T—Mo5—O13B	94.0 (2)	Co2—O9C—H9	124 (6)
O23T—Mo5—O13B	96.80 (19)	Mo7—O9C—H9	107 (6)
O14B—Mo5—O13B	149.63 (17)	Mo8—O9C—H9	121 (6)
O24T—Mo5—O8C	97.73 (19)	Co2—O10C—Mo9	102.40 (18)
O23T—Mo5—O8C	156.16 (18)	Co2—O10C—Mo8	102.77 (17)
O14B—Mo5—O8C	73.36 (16)	Mo9—O10C—Mo8	93.47 (15)
O13B—Mo5—O8C	79.06 (16)	Co2—O10C—H10	114 (6)
O24T—Mo5—O2D	163.6 (2)	Mo9—O10C—H10	127 (6)
O23T—Mo5—O2D	86.08 (18)	Mo8—O10C—H10	113 (6)
O14B—Mo5—O2D	85.50 (17)	Mo2—O11B—Mo1	118.5 (2)
O13B—Mo5—O2D	73.24 (15)	Mo3—O12B—Mo2	117.0 (2)
O8C—Mo5—O2D	70.21 (14)	Mo4—O13B—Mo5	114.7 (2)
O32T—Mo7—O31T	105.8 (2)	Mo5—O14B—Mo1	117.2 (2)
O32T—Mo7—O26B	101.1 (2)	Mo6—O25B—Mo7	117.8 (2)
O31T—Mo7—O26B	98.9 (2)	Mo8—O26B—Mo7	117.8 (2)
O32T—Mo7—O25B	95.7 (2)	Mo8—O27B—Mo9	118.4 (2)
O31T—Mo7—O25B	102.3 (2)	Mo10—O28B—Mo9	117.6 (2)
O26B—Mo7—O25B	148.24 (17)	H1A—O1W—H1B	144 (10)
O32T—Mo7—O9C	95.2 (2)	H2A—O2W—H2B	118.6
O31T—Mo7—O9C	158.44 (19)	H3A—O3W—H3B	109.5
O26B—Mo7—O9C	71.65 (17)	H4A—O4W—H4B	109.5
O25B—Mo7—O9C	80.17 (17)	H5A—O5W—H5B	108.4
O32T—Mo7—O6D	162.4 (2)	H6A—O6W—H6B	119.8
O31T—Mo7—O6D	90.14 (19)	H7A—O7W—H7B	99.6
O26B—Mo7—O6D	83.51 (17)	H8A—O8W—H8B	109.5
O25B—Mo7—O6D	73.08 (16)	H9A—O9W—H9B	111.5
O9C—Mo7—O6D	69.90 (14)	H10A—O10W—H10B	117.6
O34T—Mo8—O33T	106.0 (2)	H11A—O11W—H11C	109.5
O34T—Mo8—O27B	101.2 (2)	H12A—O12W—H12B	94 (6)
O33T—Mo8—O27B	98.2 (2)		

Symmetry codes: (i) −*x*, *y*−1/2, −*z*+1; (ii) −*x*+1, *y*−1/2, −*z*+1; (iii) −*x*, *y*−1/2, −*z*; (iv) *x*, *y*, *z*+1; (v) −*x*+1, *y*+1/2, −*z*+1; (vi) *x*+1, *y*, *z*.

### Hydrogen-bond geometry (Å, °)

**Table:**

*D*—H···*A*	*D*—H	H···*A*	*D*···*A*	*D*—H···*A*
O7C—H7···O30T	0.79 (4)	2.16 (5)	2.891 (6)	152 (8)
O8C—H8···O9W	0.85 (4)	2.26 (6)	2.972 (10)	141 (7)
O9C—H9···O8W^vii^	0.82 (4)	2.21 (6)	2.935 (9)	147 (8)
O10C—H10···O21T	0.80 (4)	2.27 (6)	2.932 (6)	141 (8)
O1W—H1A···O12W^viii^	0.92 (7)	1.97 (9)	2.809 (8)	151 (9)
O1W—H1B···O31T	0.87 (8)	1.99 (8)	2.803 (7)	154 (10)
O2W—H2A···O11W^v^	0.99	1.75	2.724 (8)	171
O2W—H2B···O12W^viii^	0.96	2.03	2.951 (9)	161
O3W—H3A···O38T^v^	0.96	2.55	3.055 (8)	113
O3W—H3B···O34T^viii^	0.96	2.35	2.936 (7)	119
O4W—H4A···O20T^ix^	0.96	2.10	2.876 (7)	137
O4W—H4B···O27B^ix^	0.96	2.07	2.725 (7)	124
O5W—H5A···O13W	0.97	1.79	2.682 (9)	151
O5W—H5B···O34T^viii^	0.97	1.87	2.768 (7)	153
O6W—H6A···O37T^v^	0.77	2.15	2.880 (7)	161
O6W—H6B···O33T^ix^	0.76	2.05	2.761 (7)	156
O7W—H7A···O30T^i^	0.94	2.38	2.991 (8)	122
O7W—H7B···O18T	0.82	2.27	2.915 (9)	136
O8W—H8A···O32T^x^	0.96	2.05	2.950 (9)	156
O8W—H8B···O6W^ii^	0.96	2.47	3.105 (12)	123
O9W—H9A···O24T	0.94	2.10	2.955 (9)	151
O9W—H9A···O32T^x^	0.94	2.31	2.817 (10)	113
O9W—H9B···O33T^x^	0.80	2.08	2.861 (9)	163
O10W—H10A···O7W^vi^	0.77	2.08	2.771 (12)	150
O11W—H11A···O26B^iv^	0.96	1.95	2.770 (7)	142
O11W—H11C···O11B	0.96	1.94	2.824 (7)	152
O12W—H12A···O28B	0.88 (5)	1.97 (5)	2.819 (8)	161 (11)
O12W—H12B···O16T^ii^	0.94 (5)	2.30 (9)	3.083 (9)	140 (10)

Symmetry codes: (vii) *x*−1, *y*, *z*−1; (viii) −*x*+1, *y*+1/2, −*z*; (v) −*x*+1, *y*+1/2, −*z*+1; (ix) −*x*, *y*+1/2, −*z*; (i) −*x*, *y*−1/2, −*z*+1; (x) *x*+1, *y*, *z*+1; (ii) −*x*+1, *y*−1/2, −*z*+1; (vi) *x*+1, *y*, *z*; (iv) *x*, *y*, *z*+1.
